# Is nonstructural bone graft useful in surgical treatment of lumbar spinal tuberculosis? A retrospective case–control study

**DOI:** 10.1097/MD.0000000000004677

**Published:** 2016-09-02

**Authors:** Jia-Ming Liu, Xuan-Yin Chen, Yang Zhou, Xin-Hua Long, Wen-Zhao Chen, Zhi-Li Liu, Shan-Hu Huang, Hao-Qun Yao

**Affiliations:** Department of Orthopaedic Surgery, The First Affiliated Hospital of Nanchang University, Nanchang, PR China.

**Keywords:** combined approach, lumbar tuberculosis, nonstructural bone, posterior approach

## Abstract

Surgical intervention is an important option for treating spinal tuberculosis. Previous studies have reported different surgical procedures and bone grafts for it. To our knowledge, few studies demonstrated the clinical results of using nonstructural autogenous bone graft in surgical treatment of spinal tuberculosis.

The purpose of this study is to compare the clinical outcomes of surgical management lumbar spinal tuberculosis by one-stage posterior debridement with nonstructural autogenous bone grafting and instrumentation versus anterior debridement, strut bone grafting combined with posterior instrumentation.

A total of 58 consecutive patients who underwent surgical treatment due to lumbar spinal tuberculosis from January 2011 to December 2013 were included. A total of 22 patients underwent one-stage posterior debridement, nonstructural autogenous bone grafting, and instrumentation (group A), and 36 patients received anterior debridement, strut bone grafting combined with posterior instrumentation (group B). The operative duration, total blood loss, perioperative transfusion, length of hospital stay, hospitalization cost, and complications were recorded. The bony fusion of the graft was assessed by computed tomography scans. American Spinal Injury Association (ASIA) Impairment Scale was used to evaluate the neurological function of patients in the 2 groups.

All the patients were followed up, with a mean follow-up duration of 21.6 ± 5.7 months in group A and 22.3 ± 6.2 months in group B (*P* = 0.47). The average operative duration was 257.5 ± 91.1 minutes in group A and 335.7 ± 91.0 minutes in group B (*P* = 0.002). The mean total blood loss was 769.6 ± 150.9 mL in group A and 1048.6 ± 556.9 mL in group B (*P* = 0.007). Also, significant differences were found between the 2 groups in perioperative transfusion volumes, length of hospital stay, and hospitalization cost (*P* < 0.05), which were less in group A compared with group B. Patients with ASIA grade C/D in the 2 groups were improved with 1 to 2 grades after the surgery with no statistical difference (*P* = 1.000). The perioperative complications rate was 9.1% (2/22) in group A and 13.9% (5/36) in group B (*P* = 0.897).

Based on a retrospective study, the procedure of one-stage posterior debridement, nonstructural autogenous bone grafting, and instrumentation has a significant shorter operative duration, lower blood loss and perioperative transfusion, shorter hospital stay, and less hospitalization cost compared with the one of anterior debridement, strut bone grafting combined with posterior instrumentation for treating lumber spinal tuberculosis.

## 1 Introduction

Spinal tuberculosis is a common disease in current clinical practice, and it accounts for nearly 50% of the cases of musculoskeletal tuberculosis.^[1]^ Lower thoracic and lumbar spine are the most common regions that the infection affected.^[2]^ Although antituberculous chemotherapy is still the main treatment for it, surgical intervention plays a more and more important role in treating it during the last decade.^[3,4]^ The surgical approaches for spinal tuberculosis include anterior, combined anterior and posterior, and one-stage posterior procedure. Anterior debridement and strut bone grafting combined with posterior instrumentation is an effective procedure for the treatment of spinal tuberculosis with kyphosis deformity.^[5,6]^ However, several studies have reported the disadvantages of this surgical procedure, such as longer operation duration and hospital stay, greater intraoperative blood loss, and higher rate of complications compared with single anterior or posterior approach.^[7,8]^ Thus, more and more spine surgeons performed one-stage posterior debridement, bone grafting, and instrumentation for spinal tuberculosis and received satisfactory clinical outcomes in patients.^[9–11]^ However, to our knowledge, few studies reported using nonstructural autogenous bone graft in surgical treatment of spinal tuberculosis with single posterior approach.

The purpose of this retrospective study is to compare the clinical outcomes of surgical treatment of lumber spinal tuberculosis by one-stage posterior debridement, nonstructural autogenous bone grafting, and instrumentation versus anterior debridement, strut bone grafting, and posterior instrumentation.

## 2 Materials and methods

This study was approved by the Ethics Board Committee of our hospital, and informed consents were received from all the patients. A total of 58 consecutive patients, who underwent surgical treatment due to lumbar spinal tuberculosis from January 2011 to December 2013, were included in this study. All the medical records and radiographic films of the patients were retrospectively reviewed. The diagnosis of lumbar tuberculosis was made according to the patients’ symptoms, laboratory tests of erythrocyte sedimentation rate (ESR), C-reactive protein (CRP), and radiographic findings obtained from plain film, computed tomography (CT), normal or contrast enhanced magnetic resonance imaging (MRI). All the diagnoses were confirmed by histological examination after the surgery. Patients were admitted to our hospital with the symptoms of lower fever, night sweats, weakness, weight loss, low back pain, and local tenderness. Some patients presented with local kyphosis deformity of lumbar spine resulting from delayed treatment.

Of the patients included, 22 underwent one-stage posterior debridement, nonstructural autogenous bone grafting, and instrumentation (group A), and 36 received anterior debridement, strut bone grafting combined with posterior instrumentation (group B). Preoperative variables of the 2 groups are demonstrated in Table 1. No significant differences were found between groups A and B for patients’ gender, age, tuberculous focus locations, and comorbidities (Table 1). The average preoperative ESR and CRP in groups A and B were 53.6 ± 25.9 versus 61.3 ± 21.7 mm/h and 34.4 ± 30.2 versus 27.5 ± 19.9 mg/L, respectively. There were no significant differences between the 2 groups (Table 2).

**Table 1:**
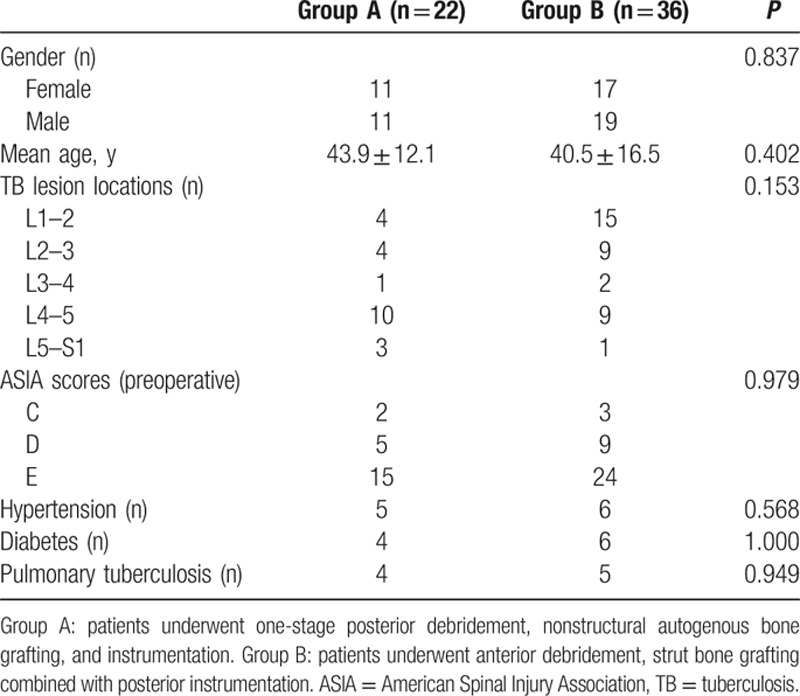
Demographic characteristics of patients in groups A and B.

**Table 2:**
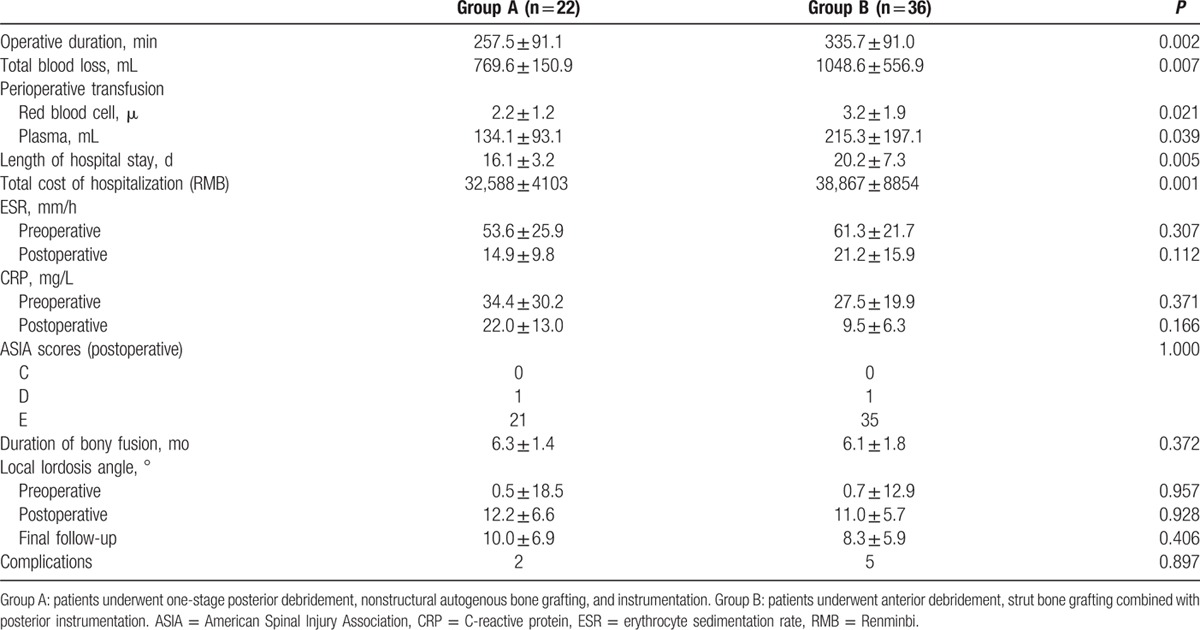
Clinical outcomes of the 2 surgical groups for lumbar spinal tuberculosis.

Inclusion criteria were patients diagnosed as lumbar spinal tuberculosis with continuous low back pain, neurological deficit, kyphosis deformity, and those who failed to standard antituberculous chemotherapy. Patients who presented with skipped lesion of lumbar spine and active pulmonary tuberculosis were excluded.

### 2.1 Preoperative management

Patients participating in both groups A and B were all administrated with the HREZ chemotherapy regimen for at least 2 weeks before surgery. The regimen consisted of rifampicin (450 mg/d), isoniazid (300 mg/d), ethambutol (750 mg/d), and pyrazinamide (750 mg/d). The surgery was performed until ESR and CRP significantly decreased (ESR < 40 mm/h),^[12]^ and tuberculosis toxicity symptoms obviously improved.

### 2.2 Surgical procedures

**Figure 1:**
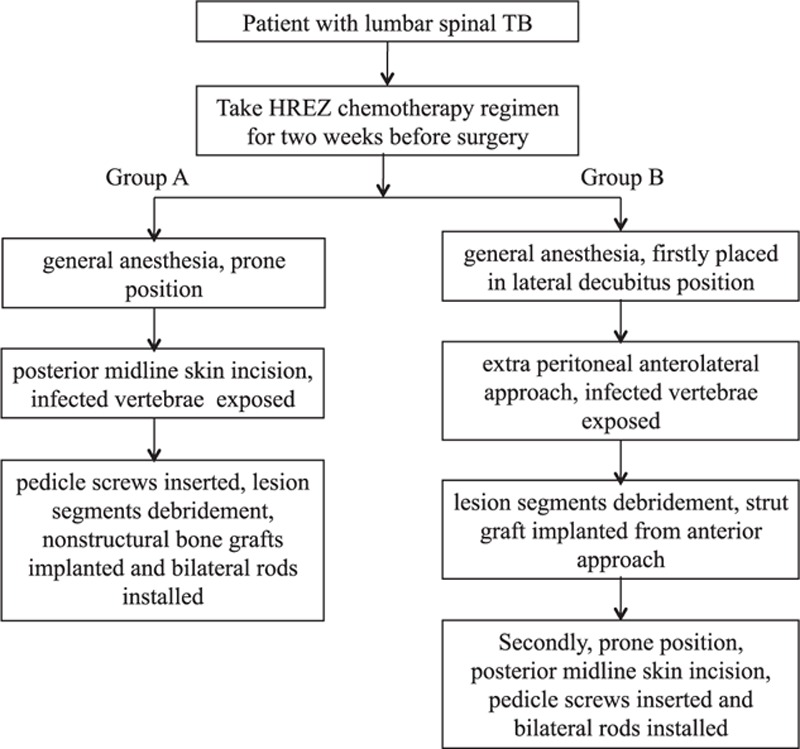
The process of preoperative management and surgical procedures in groups A and B.

In group A, patients were placed in the prone position after administration of general anesthesia. A midline skin incision of the back was done, and the posterior elements of the infected vertebrae were exposed. Posterior pedicle screws were inserted at 1 or 2 levels superior and inferior to the level of debridement. The affected vertebrae were also fixed with pedicle screws if the upper part of the vertebral body was not destroyed by infection. A temporary rod was usually used to prevent spinal nerve root injury during posterior decompression. The posterior lumbar interbody fusion^[10]^ or transforaminal lumbar interbody fusion procedure^[13]^ was performed according to the locations of the cold abscess and the lesion of the vertebrae. The lesion segments were debrided, and the cold abscesses were completely removed. If patients were associated with local kyphosis, a deformity correction was performed by installing contoured rods with compression maneuvers. The corticocancellous chips (nonstructural bone grafts) obtained from the local bone were implanted into the interbody space. Sometimes, autogenous cancellous bone chips (nonstructural bone) were harvested from posterior iliac crest if bone graft substrate was not enough. Finally, the drainage was performed, and the incision was closed (Fig. 1).

In group B, patients were first placed in the lateral decubitus position. An extra peritoneal anterolateral approach was made, and the infected vertebrae and paravertebral abscesses were exposed. The lesion of the vertebrae and the abscesses were completely debrided. Then, a strut graft harvested from tricortical iliac bone was implanted into the interbody space to reconstruct the anterior column of the lumbar spine. Second, patients were moved to a prone position, and a midline incision of the back was conducted. After exposing the posterior elements, posterior pedicle screws were placed at 1 or 2 levels superior and inferior to the level of lesion. In addition, bilateral contoured rods were installed to correct the kyphosis deformity. Finally, anterior and posterior drainages were performed after surgery (Fig. 1).

The operative duration, total blood loss, perioperative transfusion, length of hospital stay, total cost of hospitalization, and perioperative complications were recorded.

### 2.3 Postoperative management

Postoperatively, the drainage tubes were removed within 72 hours when drainage volume was less than 50 mL/24 h. In both the groups, patients were allowed to walk by themselves with a waist brace 2 weeks after the surgery. All patients received antituberculous chemotherapy for another 9 to 12 months postoperatively. The regimen included 4 drugs (rifampicin, isoniazid, ethambutol, and pyrazinamide) for 2 months followed by 3 drugs (rifampicin, isoniazid, and ethambutol) for the rest of period.

### 2.4 Follow-ups

The patients in both the groups were followed up at an interval of 3 months in the first year. Then, it was done at 6-month intervals. During the follow-up period, ESR and CRP were tested at each visit, and plain films and CT scans were conducted to evaluate the bony fusion of the implanted bone graft in the 2 groups. Evidences for bony fusion were defined as the presence of trabecular bone bridging between the bone grafts and the vertebrae on CT sagittal planes. The local lumbar lordosis angle was measured on the lateral plain film using Cobb method. Preoperative and postoperative neurological function of patients in the 2 groups was assessed by American Spinal Injury Association (ASIA) Impairment Scale.

### 2.5 Statistic analysis

All data are presented as mean ± standard deviation. The *t* test was used to analyze the continuous variables between the 2 groups. Categorical data were analyzed with the chi-square test. All the data were statistically analyzed with SPSS19.0 software (SPSS Inc., Chicago, IL). Values of *P* < 0.05 were considered to be significant difference.

## 3 Results

All the patients were followed up for at least 12 months, with a mean follow-up duration of 21.6 ± 5.7 months in group A and 22.3 ± 6.2 months in group B (*P* = 0.47). The average operative duration were 257.5 ± 91.1 minutes in group A and 335.7 ± 91.0 minutes in group B, and significant difference was found between the 2 groups (*P* = 0.002). The mean total blood loss was 769.6 ± 150.9 mL in group A and 1048.6 ± 556.9 mL in group B, which was significantly greater in the latter (*P* = 0.007). The average perioperative transfusion volumes, length of hospital stay, and cost of hospitalization were significantly larger in patients of group B than those of group A (Table 2).

Both of the ESR and CRP were significantly decreased in groups A and B after the surgery. The postoperative ESR and CRP were 14.9 ± 9.8 versus 21.2 ± 15.9 mm/h and 22.0 ± 13.0 versus 9.5 ± 6.3 mg/L in groups A and B, respectively. In addition, no significant differences were noted between the 2 groups (*P* = 0.112 and 0.166) (Table 2).

**Figure 2:**
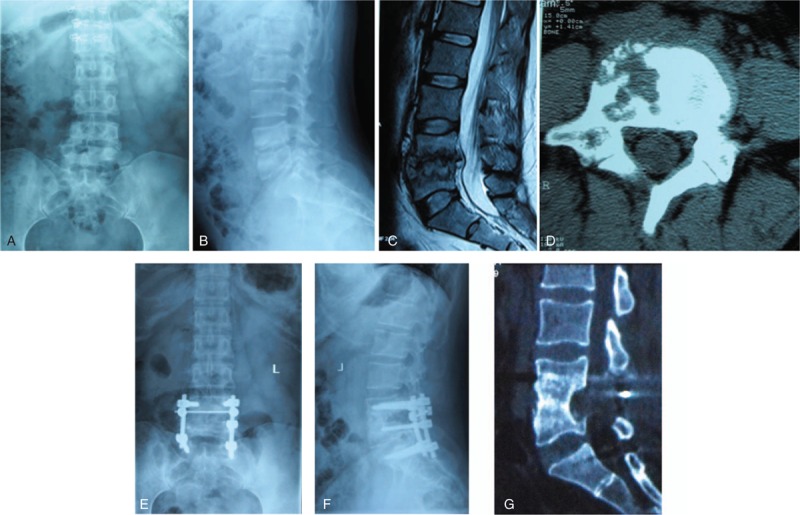
A 32-year-old female patient was diagnosed as spinal tuberculosis at the segments of L4 to L5 with paravertebral abscess. (A and B) Preoperative X-ray films of the lumbar spine. (C) Preoperative computed tomography (CT) shows vertebral destruction. (D) Preoperative magnetic resonance imaging sagittal plane shows L4 and L5 vertebral bodies bone lesion and paravertebral abscess. (E and F) The patient underwent one-stage posterior debridement with nonstructural autogenous bone grafting and instrumentation. (G) The CT scan at 12 months follow-up visit, which shows successful interbody bony fusion.

**Figure 3:**
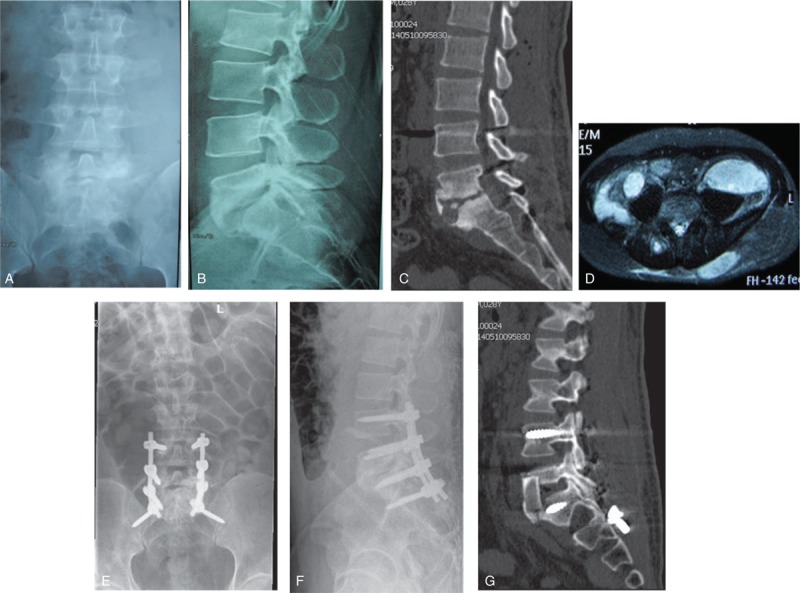
A 29-year-old male patient presented with lumbosacral spinal tuberculosis. (A and B) Preoperative X-ray films of the lumbar spine. (C) Preoperative computed tomography (CT) sagittal films shows significant bone lesion on L3 and L4 vertebral bodies. (D) Preoperative magnetic resonance imaging shows huge prevertebral and abdominal cold abscess. (E and F) The patient underwent combined anterior debridement, strut bone grafting with posterior instrumentation. (G) The CT sagittal construction film of lumbosacral spine at 1 month postoperatively.

All patients in the 2 groups presented with the evidences of successful interbody bony fusion during the follow-up. The mean duration of bony fusion were 6.3 ± 1.4 months in group A and 6.1 ± 1.8 months in group B, which was not statistically different (*P* = 0.372). The postoperative local lordosis angles in the 2 groups were increased compared with the preoperative values, and they got a little loss at the final follow-up. However, no statistical difference was detected between the 2 groups (*P* = 0.406) (Table 2). Illustrated patient cases are showed in Figs. 2 and 3.

Neurological outcomes of groups A and B are demonstrated in Table 3. Patients with ASIA grade C/D in the 2 groups improved 1 to 2 grades after the surgery. No significant statistical difference was found between the 2 groups postoperatively (*P* = 1.000) (Table 2).

**Table 3:**
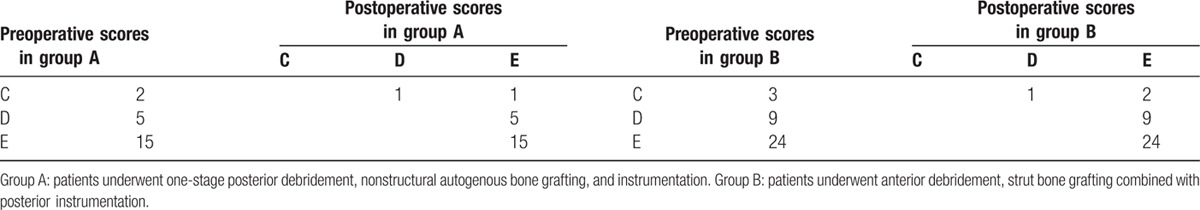
Neurologic outcomes according to ASIA scale in groups A and B.

The perioperative complications rates were 9.1% (2/22) in group A and 13.9% (5/36) in group B (*P* = 0.897). Of these patients, 2 in group A and 1 in group B had wound infection, who responded well to early debridement and antibiotics. One patient in group B got paralytic ileus 1 day after surgery and relieved by gastrointestinal decompression. Three patients in group B had bone donor site pain, which disappeared at 3 months follow-up visit (Table 3). No neurological complications or implants failure were observed in either group.

## 4 Discussion

Spinal tuberculosis is a common disease in the developing countries. Although the rate of conservative treatment of spinal tuberculosis significantly increases after the using of antituberculous chemotherapy during the past decade, more and more patients required surgical intervention because of presenting kyphosis deformity, neurologic deficit, massive paravertebral cold abscesses, and bad response to chemotherapy.^[14]^ Previous studies have reported several different procedures for surgical treatment of spinal tuberculosis.^[3,4]^ Anterior debridement with strut bone grafting is the gold standard surgical management for spinal tuberculosis,^[15]^ but it may reduce the biomechanical stability of the spine, and it is common to find residual kyphosis at the end of treatment.^[16]^ A combined anterior and posterior approach to fixation helps overcome stability-related drawbacks with the anterior approach used alone and got popular recently.^[17,18]^ However, longer operation time, greater blood loss, and double the surgical trauma for patients comparing to the anterior approach were reported in this approach. Recently, one-stage posterior approach is widespreadly used for spinal tuberculosis.^[5,6,9–11]^ However, the bone grafts used in previous studies are mainly structural bone harvested from iliac crest, rib, and fibula. Some surgeons also used mesh cage to reconstruct the anterior column of spine.^[19,20]^ Few studies reported about using nonstructural autogenous bone graft in treating spinal tuberculosis. To our knowledge, this retrospective study is the first one to compare the clinical outcomes of one-stage posterior debridement with nonstructural autogenous bone grafting and instrumentation versus combined anterior and posterior approaches in surgical management of lumbar tuberculosis.

Several previous studies have reported the comparison of single posterior versus anteroposterior approaches for treating spinal tuberculosis. A study carried out by Wang et al^[21]^ showed that patients with thoracic and lumbar spinal tuberculosis who underwent single posterior procedure had a shorter operating time, less intraoperative bleeding, and a shorter time hospital stay than those who received combined anterior and posterior procedure. Another study compared the clinical outcomes of surgical management by one-stage posterior approach and combined posterior and anterior approaches for lumbar spinal tuberculosis, and indicated that the average operative duration and blood loss in the former was less than those in the latter.^[22]^ In line with previous studies, the operative duration, blood loss, perioperative transfusion, length of hospital stay, and hospitalization cost in group A were significantly less compared with group B in the present study. However, the bone graft used in Wang et al^[21]^ study was strut bone, and in Zhang et al^[22]^ study was titanium mesh cage filled with bicortical iliac bone. Different from the previous studies, we used nonstructural autogenous bone as bone graft in this study.

The nonstructural bone grafts used in our study were harvested from the local bone chips by decompression and morselized cancellous bone obtained from posterior iliac crest. Compared to strut bone and mesh cage, nonstructural autogenous bone graft had several advantages in surgical treatment of spinal tuberculosis. At first, it is more convenient, and with less iatrogenic injury, to get nonstructural bone compared with strut iliac bone.^[23,24]^ At second, no extensive exposure is needed when implanting it into the interbody space, which would decrease the risk of nerve root injury during posterior surgery.^[25]^

In the present study, all the patients from the 2 groups got bony fusion after the surgery. In addition, the duration of bony fusion in group A was similar to group B (6.3 ± 1.4 vs 6.1 ± 1.8 months), which demonstrated that the nonstructural bone had the same union ability compared with strut bone. Zeng et al^[26]^ compared single-stage posterior surgery with a combined posterior–anterior surgical approach for the treatment of adults with lumbosacral spinal tuberculosis and found that all patients achieved bone fusion within 5 to 11 months after surgery. Also, the lumbosacral angle was significantly corrected postoperatively, with a little loss at final follow-up. Zhang et al^[27]^ conducted a study to compare the clinical outcomes of surgical management by posterior only and combined posterior and anterior approaches for thoracic spinal tuberculosis. The results showed that the average fusion duration was 7.9 months in the 2 groups, and there were no significant differences in thoracic angle correction and loss between the 2 groups. In accordance with these studies, the lumbar lordosis angles were corrected after surgery in both the groups with no significant difference in our study.

For neurological function, 77.3% of patients in single posterior approach group and 80% in anteroposterior approach group were returned to normal after surgical treatment of thoracic and lumbar spinal tuberculosis in Wang et al^[21]^ study. In Zhang et al^[22]^ study, the neurological status of 37 patients underwent either one-stage posterior procedure or combined posterior and anterior approaches were improved with 1/2 grades of Frankel scale at the last follow-up visit. Similar to these studies, the neurological outcomes in the 2 groups of our study were also improved after the surgery, and no significant different was noted. It indicated the procedure of one-stage posterior debridement with nonstructural bone grafting created the similar clinical outcomes to combined anterior and posterior procedure for treating lumbar tuberculosis.

Spine surgeon may worry about the complications related to nonstructural bone grafting used in the treatment of lumbar tuberculosis, such as bone graft subsidence, collapsing, implant failure, lordosis correction loss, and so on. However, we have received satisfactory clinical outcomes in both the groups. The rates of perioperative complications in the 2 groups were 9.1% and 13.9% (*P* = 0.897), respectively. In addition, no implant failure or bone graft subsidence was detected in group A. In Zeng et al^[26]^ study, 21.1% (4/19) of patients in single posterior approach group and 50% (10/20) in combined approach group got postoperative complications, which were higher than those of our study. Liu et al^[8]^ conducted a meta-analysis to evaluate the clinical outcomes of posterior versus combined posterior and anterior approach for treatment of spinal tuberculosis and concluded the complications rate of the former was significantly less than that of the latter. Though there was no significant difference for the complications between the 2 groups in our study, the rate of complications in single posterior group was a little lower compared with that of combined group.

Although satisfactory clinical outcomes were demonstrated in this study, several limitations were presented in it. For example, only small sample size and short-term follow-up were reported in both the groups. In addition, it was a retrospective study and the choice of one-stage posterior approach or combined anterior and posterior approaches was based on the discussion of surgeon with patients, which may affect the clinical outcomes of the 2 groups. Thus, we believe that a randomized control study with a larger sample size and long-term follow-up is needed to improve the level of the evidence for this study.

In conclusion, based on a retrospective study, the procedure of one-stage posterior debridement, nonstructural autogenous bone grafting, and instrumentation has a significant shorter operative duration, lower blood loss and perioperative transfusion, shorter hospital stay, and less hospitalization cost than those of combined anterior debridement, strut bone grafting with posterior instrumentation for the treatment of lumber spinal tuberculosis, though a randomized control study with a larger sample size and long-term follow-up is needed to determine the long-term outcomes.
